# Value associations bias ensemble perception

**DOI:** 10.3758/s13414-019-01744-1

**Published:** 2019-05-08

**Authors:** Daniel B. Dodgson, Jane E. Raymond

**Affiliations:** School of Psychology, University of Birmingham, Edgbaston, Birmingham UK

**Keywords:** Reward, Ensemble perception, Scene perception, Attention, Working memory

## Abstract

Ensemble perception refers to awareness of average properties, e.g. size, of “noisy” elements that often comprise visual arrays in natural scenes. Here, we asked how ensemble perception might be influenced when some but not all array elements are associated with monetary reward. Previous studies show that reward associations can speed object processing, facilitate selection, and enhance working-memory maintenance, suggesting they may bias ensemble judgments. To investigate, participants reported the average element size of brief arrays of different-sized circles. In the learning phase, all circles had the same color, but different colors produced high or low performance-contingent rewards. Then, in an unrewarded test phase, arrays comprised three spatially inter-mixed subsets, each with a different color, including the high-reward color. In different trials, the mean size of the subset with the high-reward color was smaller, larger, or the same as the ensemble mean. Ensemble size estimates were significantly biased by the high-reward-associated subset, showing that value associations modulate ensemble perception. In the test phase of a second experiment, a pattern mask appeared immediately after array presentation to limit top-down processing. Not only was value-biasing eliminated, ensemble accuracy improved, suggesting that value associations distort consciously available ensemble representation via late high-level processing.

## Introduction

The world is rich with visual redundancy; in most natural scenes, similar objects occur many times simultaneously. To enhance processing efficiency and to overcome tight capacity limitations of high-level visual processing, including attention and working memory (Cohen, Dennett, & Kanwisher, 2016), evidence suggests that the brain may represent such repeated object information as a single code or *ensemble* representation rather than retaining individual object representations (Alvarez & Oliva, 2008; Ariely, 2001; Chong & Treisman, 2003, 2005; Dakin & Watt, 1997; Haberman, Brady, & Alvarez, 2015). For example, when viewing a tree, the brain may represent leaf size by calculating a statistical average of all leaf sizes visible on the tree and then use and store only this abstracted ensemble information in subsequent cognitive processing.

Ariely (2001) first demonstrated that the visual system could compress complex scenes into a summary of the scene’s statistical properties. He presented observers with a brief (500-ms) array of up to 16 heterogeneously sized circles; the task was to judge whether a subsequently presented probe circle was larger or smaller than the average size of the circles making up the original array. Using this method, he reported that mean circle size was estimated remarkably precisely, i.e., within approximately 4% of the actual average element size. Interestingly, participants were unable to correctly identify whether a specific probe item had been a member of the original set, suggesting they lacked conscious access to individual element size. This study demonstrated that even with set sizes well beyond what is considered the capacity for selective attention and working memory (WM) of around four items (Luck & Vogel, 1997; Pylyshyn & Storm, 1988), the visual system is proficient at coding and synthesizing information, even without high-level representation of individual items. Subsequent studies using similar methodologies showed evidence of statistical averaging for other low-level features, including element orientation (Dakin & Watt, 1997; Haberman et al., 2015), color (Haberman et al., 2015), speed and direction of motion (Alvarez & Oliva, 2008; Sweeny, Haroz, & Whitney, 2012), and size (Chong & Treisman, 2003, 2005).

One account of ensemble perception is that only a subset of elements is actively selected and that ensemble statistics are calculated solely on this subset using high-level WM-related processes (Myczek & Simons, 2008). However, arguments against this view are substantial (Cohen et al., 2016), and were first advanced by Triesman’s group who showed that accuracy of ensemble judgments is largely unaffected by array size (Chong & Treisman, 2003, 2005), a finding consistent with the notion of a fast, pre-selective, parallel scene analyser (Oliva & Torralba, 2006; Treisman & Gelade, 1980). Other studies showed that increasing array size could even improve performance (Ariely, 2001; Robitaille & Harris, 2011), a manipulation that should degrade performance if ensemble perception were based on subset selection and WM. Additional evidence against the involvement of slow selective attention processes is that accurate ensemble estimates are possible with stimulus durations as brief as 50 ms (without pattern masks; Chong & Treisman, 2003) or 200 ms (with pattern masks; Whiting & Oriet, 2011), durations that are shorter than that required to shift selective attention from one element to another (Egeth & Yantis, 1997). Furthermore, Im and Halberda (2013) systematically manipulated the physical variability of array items and then applied variance summation modeling to demonstrate that the effects of internal noise and sampling on ensemble estimation are more akin to that seen for texture processing than for multiple individual items processing. Lastly, several studies have shown that ensemble perception of high-level properties, including average emotion expressed in faces (Haberman & Whitney, 2007) and average numeric value of numbers (Corbett, Oriet, & Rensink, 2006) can be accurately reported under conditions in which individual items cannot be explicitly identified.

A more widely accepted view of ensemble perception initially put forth by Chong and Treisman (2005) is that individual scene elements automatically undergo parallel processing that is just sufficient to contribute data regarding the feature dimension required by the task to the mechanism computing the ensemble average (Brady & Alvarez, 2011; Corbett & Oriet, 2011). In this view, high-level processing mechanisms that support awareness and WM only have access to the ensemble representation, with all but a few individual elements being inaccessible (Cohen et al., 2016; Leib, Kosovicheva, & Whitney, 2016; Whitney & Leib, 2018) This restrictive view of ensemble processing suggests that element variability on task-irrelevant dimensions (e.g., element color in an ensemble size judgment task) should have little influence on ensemble representations directed at a different feature dimension (e.g., size). Indeed, evidence from an experiment by Brady and Alvarez (2011) support this possibility. Using a WM retro-cue task, they briefly presented arrays comprised of circles of different sizes; smaller than average circles were presented in one color and larger than average circles in another color. After 1 s, a probe circle was presented in the location of one of the array elements. Participants were asked to adjust its size to match the array circle just seen at that location. Interestingly, when color was irrelevant, responses were biased towards the mean size of all the circles in the array, not the mean size of the circles with the probed circle’s color. This suggests that individual element sizes were unavailable for report, forcing participants to default to the average. Importantly, variation on an irrelevant dimension (color) had no apparent impact on ensemble size perception.

Here, we explored whether ensemble perception is similarly immune to task-irrelevant, but non-sensory, i.e., learned, attributes of elements. Specifically, we asked whether subsets of elements in an array that had task-irrelevant reward associations would bias ensemble perception for the whole array. For example, would perception of average car size in a crowded parking lot be biased toward the size of a few high-value cars or would car value effectively be ignored? Numerous studies have shown that objects associated with monetary rewards are prioritized for visual processing resulting in better recognition, even when attention is limited, exposures are brief, and rewards are no longer forthcoming (O’Brien & Raymond, 2012; Raymond & O’Brien, 2009). Moreover, objects with reward-associated features are better maintained in visual working memory (Gong & Li, 2014; Thomas, FitzGibbon, & Raymond, 2016). When presented as distractors, reward-associated objects are also more likely to distract task-relevant processing (Anderson, Laurent, & Yantis, 2011a, 2011b; Hickey, Chelazzi, & Theeuwes, 2010; Maclean & Giesbrecht, 2015; Rutherford, O’Brien, & Raymond, 2010) more than similar, equally familiar yet motivationally neutral objects. Some of these behavioral studies show reward effects even with very brief, masked exposures (O’Brien & Raymond, 2012) and with arrays of more than four items (Anderson et al., 2011a), suggesting that value associations may influence processing at early parallel-processing stages. If so, then it is reasonable to expect that a subset of value-associated items in an array could influence ensemble encoding even when array exposures are brief and masked. However, other evidence argues that value associations only influence processing at later, post-attentive stages where processing is capacity limited and serial (Pedale & Santangelo, 2015; Raymond & O’Brien, 2009; Thomas et al., 2016). If so, then the presence of some value-associated items in the array might fail to modulate ensemble processing because irrelevant, individual item information may be lost once the ensemble representation is produced (Ariely, 2001). A third, hybrid possibility is that with longer processing times (i.e., without immediate presentation of a pattern mask), an ensemble representation as well as a few selected individual items can be encoded into WM allowing ensemble reports to become influenced by other WM contents (Cohen et al., 2016; Whitney & Leib, 2018; deFockert & Marchant, 2008). Considering that value-associated items gain priority for access to visual WM (Thomas et al., 2016), then this hybrid view predicts that value-associated items might bias ensemble reports but only when processing times are sufficiently long.

To investigate, we asked participants to perform two successive ensemble size judgment tasks. In each, they were required to adjust the size of a probe circle to match the average size of 12 different-sized circles presented briefly in a circular fashion around a central fixation point. In the first task, all the circles were presented in the same color. Participants were given performance feedback on every trial using rewards that varied with response accuracy. In addition, when the array had a specific color, substantially higher point values could be earned for accurate responses than when it was a different color. Points were later exchanged for cash. Following this color-reward learning phase, participants repeated the ensemble size task, but this time the array comprised three spatially intermixed subsets of circles, each defined by a different color. No rewards were forthcoming. Importantly, the four largest circles (large set), the four mid-sized circles (medium set), or the four smallest circles (small set) were presented in the high value-associated color on different trials. A control condition was also included wherein no circles had the high value-associated color.

If value associations of individual elements in the array were available relatively early in processing (prior to ensemble representation) and were able to boost neural representations, then the contribution of reward-associated elements might be more heavily weighted than non-reward-associated elements by the statistical averaging mechanism. In our experiments this should cause ensemble estimates for the entire array to be biased in favor of the value-associated subset’s size. However, if value associations are not available prior to ensemble representation, then no effect of value should be evident because all element information should become inaccessible once ensemble representations are produced. To probe how early in processing value associations might influence ensemble representations, we conducted two experiments, both using a 200-ms array presentation duration but only one (Experiment 2) presented an immediate pattern mask to stop further processing. Numerous studies show that pattern masks effectively terminate processing modulations arising from high-level feedback, preventing more stable, elaborated representations (Enns & Di Lollo, 2000; Phillips, 1974; Sligte, Scholte, & Lamme, 2009; Sperling, 1965; Vogel, Woodman, & Luck, 2006). We predicted that by presenting a pattern mask we might abolish any value-biasing effects by limiting ensemble representations to available sensory data, unaffected by previously learned information. To anticipate, we observed value-biasing of ensemble estimation when the array exposure was not masked and abolished this effect when a mask was presented.

## General method

### Participants

Sample size was based on Brady and Alvarez (2011) who tested 20 participants in a similar study and obtained an effect size of Cohen’s *d* = 1.82. A more conservative Cohen’s *d* of 1.4, power = 0.80, and a two-tailed test indicated a sample size of 19 would sufficiently power each experiment. Twenty-three participants (four males, average age = 22.0 years (SD = 4.42; range = 18–35) completed Experiment 1; a different 23 participants (three males, average age = 19.35 years (SD = 0.81, range 18–21) completed Experiment 2. All participants were recruited from the University of Birmingham, took part in exchange for course credits, or were compensated £6 (plus extra cash earned on the value-learning task). All provided informed consent prior to participation, reported normal or corrected-to-normal vision, were naïve to the purpose of the experiment, and had normal color vision as assessed using the Munsell D-15 color-blindness test.

### Apparatus

#### Value-learning and post-learning tasks

Stimulus presentation and data recording were controlled by a Macintosh computer and were programmed in Matlab (The MathWorks, Inc., Natick, MA, USA) using the Psychophysics Toolbox. Responses were recorded using a standard keyboard and mouse. Stimuli were presented in RGB color space on a black background ([0, 0, 0]) of a 68-cm LCD monitor (ASUS VG278) with a screen resolution of 1,920 × 1,080 and a refresh rate of 60 Hz. Viewing distance was approximately 60 cm.

### Stimuli

Test arrays comprised 12 color-filled circles with areas that could range between 79 pixels^2^ (diameter = 10 pixels) and 20,096 pixels^2^ (diameter = 160 pixels). The diameter of each circle in each array was drawn from a log-normal distribution (s.d. = 35 pixels). This allowed the average circle size (for the entire array) to have 25 different equally spaced values (ranging between 1,885 and 11,499 pixels^2^); each average circle area was equally probable for each array condition. Averaging across all trials and all conditions used within the session, the average circle area was 5,675 pixels^2^, which corresponds to a circle with a diameter of 1.6° of visual angle. The center of each circle was positioned randomly on the circumference of a larger central and invisible circle (radius of 8°), with the constraint that no element overlapped another. In the value-learning task, all test array circles had the same color; in the post-learning task, three different colors were used (with four circles in each color). Colors (drawn in RGB color space) used in experimental trials were purple [RGB colors 148, 131, 165], red [230, 93, 85], and orange [182, 133, 58]. Three different colors were used for control trials: pink [226, 90, 121], brown [215, 115, 58], and green [110, 151, 125].

The response display comprised a single circle presented at fixation. On initial presentation, its diameter was randomly chosen (between 10 and 160 pixels) but was never within 20 pixels of the actual mean diameter. Adjustment of circle size by the participant could lead to a minimum diameter of 10 pixels and a maximum diameter of 192 pixels. In the value-learning task, the response circle had the same color as the preceding test array; on post-learning trials it was gray (127, 127, 127).

The mask used in Experiment 2 was comprised of differently colored squares arranged in a 9.5° × 9.5° grid, each small square subtending approximately 0.5° × 0.5°. Each square was presented in one of the six possible colors used in the post-learning task. The mask was constructed on each trial by randomly selecting a color for each grid location.

Feedback displays comprised point reward values presented at the center of the screen in white on a black field in Arial font, 100 point. Running total information was positioned at the center bottom of the screen and displayed in black Arial font, 50 point.

### Procedure

#### Value-learning task

**Fig. 1:**
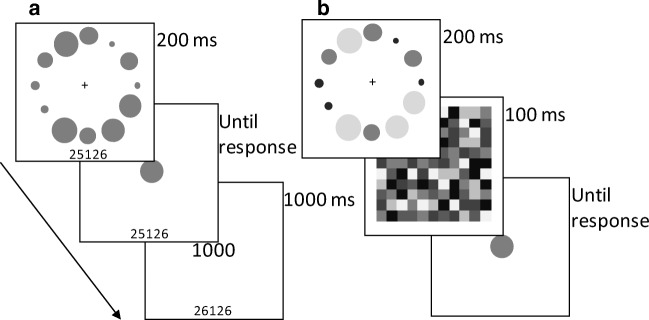
(**a**) Trial schematic for the Value-learning Task used in Experiments 1 and 2 to condition color-value associations prior to participation in the Post-Learning Task. The ensemble array was immediately followed by the response circle. Point feedback was based on response accuracy and array color. A running point total was displayed at the bottom on the screen, as shown (not to scale). (**b**) Trial schematic for the Post-learning Task as performed in Experiment 2. The sequence was similar for Experiment 1 except that the colored check pattern (mask) was not presented and the response circle was presented immediately after the offset of the stimulus array. Feedback was not provided. Gray-scale variations in the figure represent color variations in the actual display

The trial sequence is illustrated in Fig. 1a. Following a fixation cross (1,000 ms), the test array was presented for 200 ms immediately followed by the response circle (until response). After response, the number of points earned on that trial was displayed (1,000 ms). Participants were instructed to estimate the average size of all of the circles in the test array, i.e., to provide an ensemble size estimate. To indicate this, participants adjusted the size of the central response circle by moving the mouse to the left (to shrink it) or right (to enlarge it) until it matched the perceived average circle size, pressing the left mouse button to submit the ensemble estimate (cf. Brady & Alvarez, 2011). The response circle diameter adjusted linearly in response to movements of the mouse. If response was within 5% (smaller or larger) of the actual average size, the maximum number of points for that trial was presented; otherwise the percentage error (larger or smaller) was deducted from the maximum possible point award for that trial (rounded to the nearest 10%) and presented as feedback. A running total of the points earned was presented at the bottom of the screen throughout the session and was updated in the feedback display provided after each trial.

Test array circles shared one of two possible colors. One (reward) color denoted a maximum award of 1,000 points, the other (baseline) color a maximum award of 10 points. The color-outcome pairings were counterbalanced across participants. There were 200 trials in total; value and baseline trials were presented equally often in a pseudo-random manner. Participants completed four blocks of 50 trials. During inter-block breaks, participants were reminded of the number of points earned thus far and its cash equivalent (20,000 points = £1). At the end of the value-learning task, participants were given the cash that they had earned. For the first five trials of every block the participant chose the color of the test array, having been told to pick the color that would give them the highest number of points. This was used to provide an explicit measure of color-value learning. To ensure that participants had equal exposure to both colors, five extra trials were randomly placed in the subsequent block composed of the alternate color to that which the participant chose on the first five (choice) trials.

#### Post-learning test

The trial sequence illustrated in Fig. 1b was identical to that used in the value-learning task with the following exceptions. First, there was no feedback or points awarded and participants were explicitly told that no further points would be awarded. Second, the response circle was always gray. Third, in Experiment 2 only, a pattern mask was presented for 100 ms immediately following the test array offset. Fourth, test circles no longer shared the same color. The four largest circles (Large Set) were presented in one color, the four mid-sized circles (Medium Set) in another color, and the four smallest circles (Small Set) in a third color. On every trial the average size of circles in the Medium Set was equal to the average size of all circles in that array. On each trial of the experimental conditions, one set was presented in the value color, another in the baseline color, and the third in a novel color. The latter was the unused color from the color set used for the value-learning task (see Stimuli).

The experiment used a subset of the six unique combinations of color (value, baseline, novel) × set (Large, Medium, Small), omitting conditions that placed the value color and baseline color in the Small and Large set, respectively, or *vice versa.* This left the following conditions: (1) Value-Large: Novel, baseline, and value colors were used for the Small, Medium, and Large sets, respectively. (2) Value-Small: Value, baseline, and novel colors were used for the Small, Medium, and Large sets, respectively. (3) Value-Medium: The value color was used for the Medium set and the baseline and novel colors for the Small and Large sets, respectively, and *vice versa*, creating two types of Value-Medium trials. (4) Control condition: The three colors not used previously were used in the test array, one for each set. On each trial, control colors were assigned randomly to a set (without replacement). For all trials, the average circle size in the entire array always matched the average size of the Medium set.

There were 50 trials in each block: ten for each color-set combination described above plus ten control condition trials. Trial order was pseudo-random. Note: In each block, the value and baseline colors were equally likely to appear in each size-set; novel colors were never used for the medium set but were equally likely to appear in the Large or Small set. Each participant completed nine experimental blocks (450 trials). A practice block of 50 trials, followed by ten top-up learning trials (five for each value condition, pseudo-randomly presented), was given at the start of the task. Similar, brief top-up learning blocks (ten trials each) were given after blocks 3, 5, and 7 of the experimental blocks.

### Data analysis

#### Value-learning and post-learning task

Three participants in Experiment 1 and four in Experiment 2 chose the incorrect color on the choice task on more than 15% of trials, suggesting weak or absent color-value learning. All the rest of the participants performed at 100% on this easy test of learning. One participant in Experiment 1 had average misestimations in all Learning and Post-learning blocks that were more than 2 s.d.’s larger than the corresponding group means, suggesting failure to understand the task. All the data from the poor learners and from the atypically poor task performer were excluded from further analysis.

For each trial on all tasks, ensemble estimates (i.e., circle area expressed in pixels^2^) were log_10_ transformed (log_10_Est) and each circle area in the test array was similarly transformed; array circle values were then averaged to compute mean circle size (log_10_Array). Misestimation on each trial was calculated as the difference between log_10_Est and log_10_Array, where positive log unit values mean overestimation. Misestimation means were calculated for each participant and array condition and then subjected to repeated-measures analyses of variance (ANOVA), using all four or just the three value-array conditions as the within-subjects factor, as needed. Greenhouse-Geisser corrections were applied when assumptions of sphericity were violated in all ANOVAs. Paired-sample (2-tailed) t-tests were used to compare group mean misestimations and response times (RTs) for high veresus low reward trials in the learning tasks in each Experiment. Bonferroni corrections for multiple comparison were applied as needed. One-sample (2-tailed) t-tests were used establish that misestimations were different from zero. Mixed-design repeated-measures ANOVAs were used to compare results between experiments, using Experiment as a between-subjects factor and array condition as the within-subjects factor. Alpha levels were set at 0.05.

## Experiment 1

The aim of this experiment was to determine if ensemble reports of array element size would be biased by a subset of elements presented in a reward-associated color. Each array was presented for 200 ms and was never masked.

### Results and discussion

#### Value-learning task

Average ensemble misestimations and mean RT are shown in Table 1. The magnitude of ensemble misestimation in each condition was similar (*p > .5*). Circle size was significantly overestimated in both the value [*t*(18) = 6.63, *p* < .001] and the baseline [*t*(18) = 6.81, *p* < .001] conditions. However, mean RTs were 179 ms slower when the array color signalled the availability of reward versus baseline outcome [*t*(18) = 2.920, *p* = .009], suggesting greater effort in the former condition and supporting the contention that reward learning had occurred.

**Table 1:** Mean response times (RTs; ms) and misestimates (log units) of circle size in the value-learning task for baseline and value circles in Experiments 1 and 2

Trial type	Experiment 1 (No-mask)		Experiment 2 (Mask)	
	RT	Misestimation (log units)^1^	RT	Misestimation (log units)
Baseline	2497 (273)	0.183 (0.028)	2938 (240)	0.150 (.100)
Value	2670 (246)	0.178 (0.027)	3151 (271)	0.156 (.094)

Standard error of the mean is given in parenthesis

^1^Postive values indicate overestimation. See *Data analysis*

#### Post-learning

**Fig. 2:**
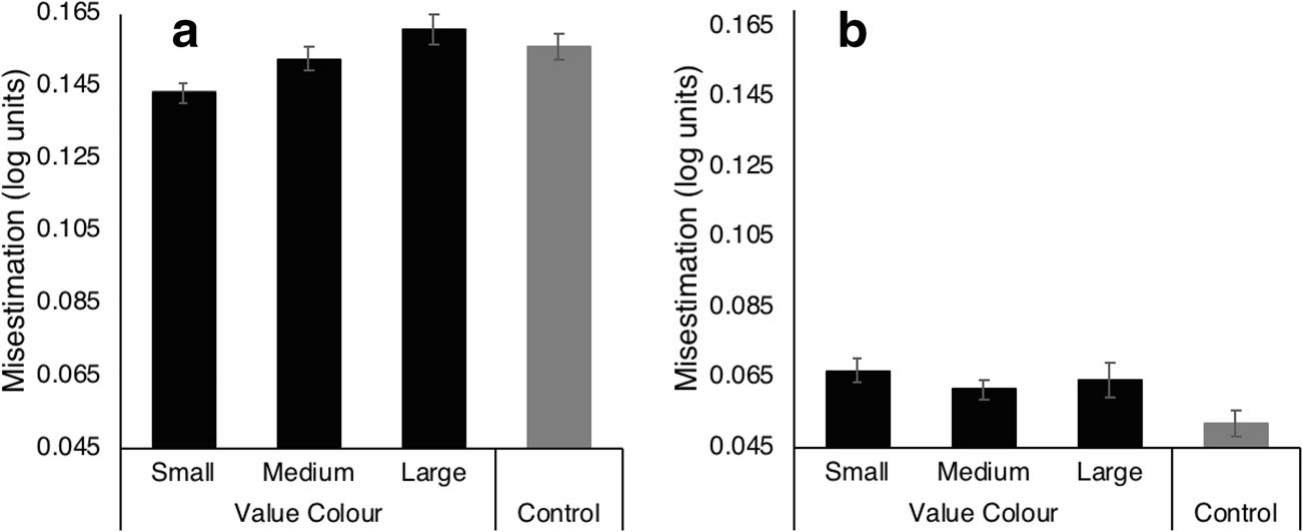
Group mean misestimation of circle size obtained in the four conditions of the test array in the Post-learning Task for Experiment 1 (**a**) and Experiment 2 (**b**). Black bars represent means when the value color appeared in the test array; gray bars when it was absent (control condition). The circle size category of the subset with the value color is indicated on the x-axis. Positive numbers represent an overestimation of the actual average circle size. Error bars reflect ±1 within-subject standard error of the mean (Cousineau, 2005)

As in the learning task, participants significantly overestimated ensemble circle area in all conditions (all *t*’s > 6, all *p’s* < .001). However, as can be seen in Fig. 2a, the magnitude of misestimation varied with array condition [*F*(2.42, 43.4) = 3.384, *p* = .035, eta^2^ = .158, power = .661]. Specifically, the presence of the value color significantly modulated ensemble estimates depending on the size-set for which it was used – the smaller the set possessing the value color, the smaller the ensemble estimate. Specifically, when the value color was used for the Small set, misestimation (mean = 0.143 log units) was significantly smaller than when this color was used for the Large set (mean = .161 log units*; t*(18) = 3.018, *p* = .007) or Medium set (mean = .152 log units; *t*(18) = 2.234, *p* = .038). Misestimation was non-significantly different between the Large and Medium size sets (*t*(18) = 1.250, *p* = .227). Ensemble misestimation obtained in the Value-Small condition was also significantly smaller than that measured in the control condition (mean = .156 log units; *t*(19)= 2.79, *p* = .011). A similar comparison for the Value-Large condition was non-significant (*p* > .45).

Experiment 1 provides novel evidence that the motivational salience of a subset of elements can bias ensemble estimation even when there is no incentive for biased selection of information from the array. When the smallest circles in the display were in the high-value-associated color, the tendency to overestimate ensemble element size was modestly but reliably reduced relative to a control (no-value) condition. Furthermore, when the large set of circles had the high-value color, ensemble estimates were reliably larger than when the same color was used for the small set.

## Experiment 2

If, in Experiment 1, motivational salience influenced ensemble estimations by modulating early perceptual representations of each element, then the introduction of a pattern mask immediately after the test array should have minimal effect on the pattern of results, i.e., the influence of the high-value colored circles should again be evident. However, if value-associations modulate processing at a later stage of processing that involves recurrent top-down processing (Enns & Di Lollo, 2000), then masking the test array might eliminate any influence of previously learned value-associations. Experiment 2 tested this by repeating the procedure of Experiment 1 but this time presenting a pattern mask immediately following the presentation of the initial ensemble test array.

### Results and discussion

#### Value-learning task

As before, reward value had no effect on ensemble estimation (*t* < 1) (see Table 1.) When misestimations from this experiment were compared to the corresponding results from Experiment 1, neither a significant main effect of experiment (*F* < 1) or reward value (F(1,37) = 1.514, p = .23, eta^2^= .039) nor an interaction (*F* < 1) was found. RTs were 213 ms longer in the value (mean = 3,151 ms) versus baseline (mean = 2,937 ms) condition (*t*(18) = 2.606, *p* = .017), replicating effects observed in Experiment 1.

#### Post-learning task

Group mean ensemble misestimations are plotted in Fig. 2b for each test array condition. In contrast to Experiment 1, array condition had a non-significant effect (*F*(2.46, 26.78) = 2.096, *p* = .124, *eta*^2^ = .099, power = .454). The linear trend of misestimation to decrease with value size set was no longer significant (*F* < 1). It is obvious from the figure that misestimates were markedly reduced in this experiment compared to Experiment 1, approaching but not reaching zero. One-way t-tests showed that in all array conditions, ensemble size was still significantly overestimated, (all *p*’s < .05).

A mixed-design ANOVA using experiment as a between-groups factor and condition as a within-subjects factor revealed a significant main effect of Experiment [*F*(1,37) = 6.067, *p* = .019, *eta*^2^ = .141, power = .670] and, importantly for the hypothesis being tested here, the interaction of Experiment and array condition was also significant [*F*(2.49,92.16) = 3.492, *p* = .025, *eta*^2^ = .086, power = .706]. The presentation of a mask after the initial test array not only eliminated any influence of motivational salience of array subsets but also substantially reduced the tendency to overestimate the average circle size in the array.

## General discussion

The aim of the study reported here was to determine whether ensemble perception could be influenced by task-irrelevant, non-sensory, i.e., learned, reward associations of elements in the array. In two experiments, participants first learned to associate element color with reward value by engaging in an element size ensemble judgment task in which all elements (circles) in the array had the same color. Then, in the test phase, the same ensemble judgment task was required, but this time arrays were multi-colored, and no rewards were forthcoming. Critically, on different trials, a subset of circles that were either larger or smaller than average had the high value color. Experiments 1 and 2 were identical except that in the latter, a pattern mask immediately followed the brief (200 ms) array presentation during the test phase. The pattern mask was used to interfere with the transfer of information to a more stable form of representation such as working memory (Enns & Di Lollo, 2000; Phillips, 1974; Sligte et al., 2009; Sperling, 1965; Vogel et al., 2006), forcing the ensemble response in the second experiment to rely on fast parallel processing. Without the mask (Experiment 1), ensemble size perception in the test phase was biased towards the mean size of the subset of circles with the high-value reward color. In addition, ensemble estimates were consistently overestimated, suggesting that the larger circles, regardless of their color, biased response. In contrast, when test arrays were pattern masked, the value-biasing effect was eliminated and the general tendency to overestimate was also reduced, although not abolished. These findings indicate that ensemble judgments are generally more accurate and less open to bias by individual element features when processing time is brief, and that under the latter conditions, low level perceptual salience of individual items can bias calculation of the ensemble statistic. However, when more time is provided, ensemble judgments are susceptible to bias by motivationally salient, i.e., reward-associated, as well as physically salient, i.e., large, items in the array.

Simple, single-mechanism explanations of ensemble perception cannot easily explain these results. Many current theories of ensemble perception propose that ensemble judgments involve rapid parallel processing of all elements in the array without separate analysis of individual items (Ariely, 2001; Chong & Treisman, 2003, 2005; Corbett & Oriet, 2011). Others argue that ensemble perception may be based on the selection and analysis of a few items from the array (Myczek & Simons, 2008) and thus involves late WM processes. Our finding that ensemble perception can be influenced by element motivational and physical salience and that such effects depend on available processing time supports a hybrid view of these two models (e.g., de Fockert & Marchant, 2008). Suppose a rapidly acquired array ensemble statistic is always prioritized for rapid representation in WM due to task demands. When additional processing time is available, one or two especially salient array elements can also be concurrently represented in WM. Explicit behavioral report in such situations may be based on a combination of these individual element representations and the ensemble statistic. In this view, masking the ensemble array, as in Experiment 2, means that the ensemble report can only be based on a singular ensemble representation, explaining why responses are more accurate and less biased. Without the mask, ensemble judgments result from WM processes that combine the rapidly acquired ensemble statistic with individual representations of elements.

Our findings indicate that physical salience as well as motivation salience heightens the likelihood of element selection when there is no mask, allowing such items to bias the final report provided by the participant. Indeed, previous studies show that both physically salient stimuli (Theeuwes, 1992) as well as value-associated stimuli (Raymond & O’Brien, 2009) garner preferential visual processing, perhaps explaining why physically salient (Pedale & Santangelo, 2015) as well as motivationally salient (Gong & Li, 2014; Krawczyk, Gazzaley, & D’Esposito, 2007; Thomas et al., 2016; Wallis, Stokes, Arnold, & Nobre, 2015) objects are better remembered than less salient objects when first encountered in natural scenes. Moreover, size contrast has been found to be an important determinant of physical salience, with larger items receiving higher visual priority (Proulx & Egeth, 2008), a finding that specifically supports our explanation of why the overestimation effects were so pronounced in Experiment 1.

It is important to note that value associations in the current study were entirely irrelevant to the size-estimation task, making their effect on ensemble judgments somewhat surprising. Previous studies have shown that variation of a task-irrelevant feature (e.g., color) in a size judgment task has little effect on performance. This was found even when array processing time was long (350 ms, unmasked) and report was delayed (Brady & Alvarez, 2011), task features that encourage active and sustained use of WM. Yet, here we show that value-associations can influence selection in an ensemble task even when task-irrelevant and indeed disadvantageous to accurate performance. Such findings are consistent with previous studies measuring responses to single targets presented within multi-item arrays (Anderson et al., 2011b). Our current finding supports the possibility that previously acquired value-associations can be used to heighten visual processing priority of specific objects or features over a wide range of situations, helping to explain why behavioral experience can shape perception in ways that cannot be predicted from available sensory data alone.

In the context of ensemble perception, value-biasing suggests that prior experience may influence our perception of larger object surface textures and scene backgrounds, as well as scene gist, as these are primary roles thought to be served by ensemble-processing mechanisms (Im & Halberda, 2013; Oliva & Torralba, 2006; Whitney & Leib, 2018). Such abstractions enable scene gist to be rapidly extracted (Oliva & Torralba, 2001) and contribute to the perception that a scene is stable after moving the eyes (Corbett & Melcher, 2014; Manassi, Liberman, Chaney, & Whitney, 2017). The current observation that ensemble perception can be value-biased underscores an emerging body of evidence that prior experience linking stimuli with motivationally salient behavioral outcomes shapes our perception of the world. If we consider that ensemble perception probably functions to provide concise descriptions of larger textured surfaces and scene backgrounds (Im & Halberda, 2013), to support rapid extraction of scene gist (Oliva & Torralba, 2006), and to contribute to the perception that a scene is stable after moving the eyes (Manassi et al., 2017), then the current observation that ensemble perception can be value-biased suggests that all these scene analysis functions are similarly susceptible to bias by prior value learning.
